# Induction of bacterial expression at the mRNA level by light

**DOI:** 10.1093/nar/gkae678

**Published:** 2024-08-10

**Authors:** Américo T Ranzani, Konrad Buchholz, Marius Blackholm, Hayat Kopkin, Andreas Möglich

**Affiliations:** Department of Biochemistry, University of Bayreuth, 95447 Bayreuth, Germany; Department of Biochemistry, University of Bayreuth, 95447 Bayreuth, Germany; Department of Biochemistry, University of Bayreuth, 95447 Bayreuth, Germany; Department of Biochemistry, University of Bayreuth, 95447 Bayreuth, Germany; Department of Biochemistry, University of Bayreuth, 95447 Bayreuth, Germany; Bayreuth Center for Biochemistry & Molecular Biology, Universität Bayreuth, 95447 Bayreuth, Germany; North-Bavarian NMR Center, Universität Bayreuth, 95447 Bayreuth, Germany

## Abstract

Vital organismal processes, including development, differentiation and adaptation, involve altered gene expression. Although expression is frequently controlled at the transcriptional stage, various regulation mechanisms operate at downstream levels. Here, we leverage the photoreceptor *Nm*PAL to optogenetically induce RNA refolding and the translation of bacterial mRNAs. Blue-light-triggered *Nm*PAL binding disrupts a *cis*-repressed mRNA state, thereby relieves obstruction of translation initiation, and upregulates gene expression. Iterative probing and optimization of the circuit, dubbed riboptoregulator, enhanced induction to 30-fold. Given action at the mRNA level, the riboptoregulator can differentially regulate individual structural genes within polycistronic operons. Moreover, it is orthogonal to and can be wed with other gene-regulatory circuits for nuanced and more stringent gene-expression control. We thus advance the pAurora2 circuit that combines transcriptional and translational mechanisms to optogenetically increase bacterial gene expression by >1000-fold. The riboptoregulator strategy stands to upgrade numerous regulatory circuits and widely applies to expression control in microbial biotechnology, synthetic biology and materials science.

## Graphical Abstract

**Graphical Abstract:**
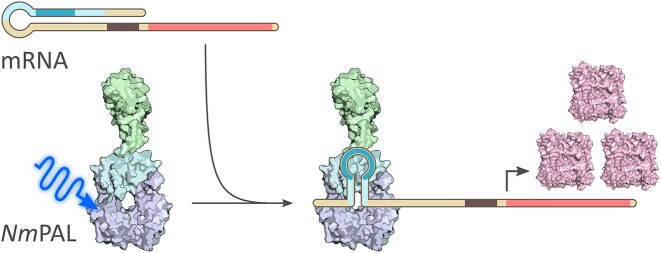

## Introduction

Differential gene expression underlies cellular state and manifold vital processes, including development, differentiation and adaptation. By necessity, the transcription of DNA into the coding mRNA and its downstream translation into polypeptide chains at the ribosome are hence subject to finely calibrated regulation mechanisms acting at multiple stages. Not least due to their comparatively simpler subcellular architecture, prokaryotes resort to a different set of gene-regulatory mechanisms than eukaryotes.

Irrespective of the exact mechanism, means of precisely and stringently regulating bacterial gene expression are also of overarching importance for various use cases in microbial biotechnology, ranging from the routine, e.g. the heterologous expression for protein production, to the more complex, e.g. diverse applications in synthetic biology and materials science (1–3). Both in nature and technology, bacterial gene expression is most commonly controlled at the level of transcription initiation, but other steps of the gene-expression trajectory have also been tapped for regulation (4). Examples include recombination events that covalently and often irreversibly alter the DNA template; transcription elongation and termination; intracellular mRNA lifetime; translation initiation, elongation, and termination; and activity state and lifetime of the expressed protein.

Many of these regulatory mechanisms play out at the mRNA level. As a case in point, certain proteins, e.g. ANTAR receptors, mediate transcriptional antitermination by binding to specific sequence and secondary-structure motifs within the nascent mRNA (5,6). The onset of translation and hence gene expression overall are governed by how readily the translation-initiation complex can be assembled at one of potentially several ribosome-binding sites (RBS) within the mRNA (7). The RBS contains the Shine-Dalgarno (SD) sequence that base-pairs with the 3′ terminus of the 16S rRNA of the 30S subunit and thereby precisely positions the ribosome relative to the start codon.

A widely used principal regulatory mechanism involves controlling the access to the RBS/SD region via modulation of the mRNA secondary structure. If this region is sequestered in a base-paired region, ribosome binding is hindered, and expression reduced. For instance, as one mode of action, the RNA-binding protein CsrA employs this strategy to gate translation initiation (8,9). Likewise, in many riboswitches the 5′-untranslated region (UTR) of the mRNA adopts different structures in the presence and absence of a cognate ligand, the RBS is hence rendered more or less accessible, and expression can be dialed up or down (10).

In a similar vein, certain small regulatory RNAs (sRNA) can base-pair to the mRNA in *trans* and thereby sequester or liberate the RBS/SD regions (11). Collins and coworkers duly recognized the potential of regulating translation initiation and gene expression by governing the access to the RBS/SD via base-pairing (12). In an approach dubbed riboregulator, choice mRNAs were decorated in their 5′-UTR with a *cis*-repressing RNA (*cis*-RNA) that looped back and thereby blocked the RBS. (Note that we depart from the abbreviation crRNA originally used by Collins to avoid confusion with the CRISPR RNA of bacterial innate immune systems.) Addition of a *trans*-activating RNA (taRNA) relieved the blockade and prompted translation. Likewise, more elaborate cascades of interacting RNA molecules allow the assembly of RNA logic gates with fine-tuned and more complex regulatory properties.

Biotechnological applications commonly require the precise control of bacterial gene expression upon delivery of a trigger signal. With light as the trigger, expression control can be exerted reversibly, noninvasively, with acute resolution in time and space, and in automatable fashion (4,13). Given these benefits, light-regulated gene expression in bacteria has gained traction and recently unlocked novel use cases (4). At the heart of these so-called optogenetic circuits for expression control lie sensory photoreceptors (14). Once stimulated by light absorption in the near-UV to near-infrared range, the photoreceptor undergoes a series of reactions, termed photocycle, and eventually adopts its signaling state that gives rise to differential expression output. Usually, the system reverts to its basal state thermally, thus rendering the light-induced response principally reversible (4). For example, light-oxygen-voltage (LOV) receptors, originally identified in land plants (15), employ flavin nucleotide chromophores to react to blue light.

As alluded to above, the most important lever for the optogenetic expression control in bacteria is transcription initiation. Apart from circuits regulating this step by second-messenger formation (16) and by directly light-regulated, single-component transcription factors (17–20), two-component systems (TCS) (21,22) sensitive to light currently dominate biotechnological applications (4). As the two most widely deployed TCSs, CcaRS from *Synechocystis* sp. (23,24) and the engineered YF1:FixJ pair (25,26) mediate the control of gene expression in response to red/green and blue light, respectively. Starting from the pDusk system, based on the YF1:FixJ TCS, we recently advanced derivative circuits that afford red-light-regulated gene expression in bacteria (27,28).

Although the optogenetic regulation of transcription initiation excels in potency and versatility, control points downstream of transcription provide viable alternatives and additions. Integrating several control layers may achieve finer-graded, multi-tiered, and stratified responses, for example for the parallel regulation of several gene products at once. Some years back, the discovery of the LOV receptor PAL in *Nakamurella multipartita* (*Nm*PAL) which sequence-specifically binds to small RNA hairpins, denoted aptamers in the following, with up to several hundred-fold enhanced affinity under blue light paved the way towards exactly such application scenarios (29).

Beyond utility for optoribogenetics in mammalian cells (29–32), *Nm*PAL also enabled the regulation of bacterial expression at the mRNA level (29,33). Within the pCrepusculo circuit (33), the PAL aptamer is interleaved with the SD such that blue-light-activated *Nm*PAL binding impedes assembly of the translation-initiation complex and thus lowers gene expression (Supplementary Figure S1). We previously extended pCrepusculo by the widely used λ phage cI repressor to achieve gene expression that is amplified and activated by blue light (33). However, the resulting pAurora circuit exhibited moderate basal expression in darkness and reduced regulatory efficiency, and it departed from purely RNA-based regulatory mechanisms.

Here, we apply *Nm*PAL and RNA refolding equilibria to optogenetically activate translation under blue light. The insertion of PAL-specific aptamers into *cis*-repressing RNAs prepended to the 5′ terminus of mRNAs upgrades the riboregulator concept to the riboptoregulator strategy. Within this strategy, blue-light-induced binding of *Nm*PAL to the aptamer relieves RBS/SD obstruction and therefore enables translation initiation and elongation. The systematic and random probing of sequence determinants identifies riboptoregulator variants with up to 30-fold upregulation of expression under blue light, thus surpassing many other approaches for regulating translation. As the riboptoregulator operates at the mRNA level, it is orthogonal to scores of gene-regulatory circuits targeting transcription and hence lends itself to integration with them. As a case in point, an upgraded pAurora circuit with integrated riboptoregulator achieves more than 10^3^-fold upregulation of target genes under blue light with very low basal activity. This circuit in particular and the riboptoregulator concept in general stand to benefit diverse applications in synthetic biology, basic research, and microbial biotechnology.

## Materials and methods

### Molecular biology

**Figure 1.**
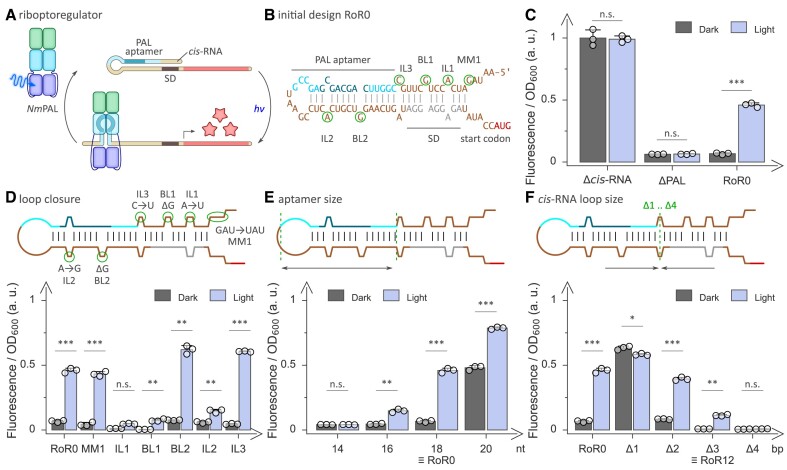
The riboptoregulator (RoR) strategy for activating bacterial gene expression at the mRNA level by blue light. (**A**) Within the RoR circuit, a *cis*-repressing RNA (*cis*-RNA) stretch prepended at the 5′ end of an mRNA loops onto the Shine-Dalgarno sequence (SD) and thereby blocks translation initiation. The *cis*-RNA comprises the motif-3 aptamer (33) that *Nm*PAL binds once exposed to blue light. The blockade is thereby relieved, and translation may ensue. (**B**) Predicted secondary structure (40) of the initial riboptoregulator design RoR0. BL, IL, and MM denote bulge loops, internal loops, and mismatches, respectively, within the folded *cis*-RNA. (**C**) Normalized *Ds*Red reporter fluorescence of bacteria harboring the RoR0 circuit when incubated in darkness (grey bars) and under blue light (blue bars), respectively. Control circuits lack either the *cis*-RNA or *Nm*PAL and consequently give rise to constitutively high and low expression, respectively. (**D**) The bulges, internal loops, and mismatches within the *cis*-RNA of the original RoR0 were closed individually, and the effect on light-dependent reporter-gene expression was assessed. (**E**) Modifications of the motif-3 aptamer length, 18 nucleotides (nt) in the original RoR0, and their effect on circuit output. (**F**) The size of the *cis*-RNA was modified by moving the motif-3 aptamer closer to the SD sequence, and the effect on reporter fluorescence in darkness and light was determined. The variant shortened by 3 base pairs (bp) and referred to as RoR12 drastically reduced basal fluorescence in darkness while retaining reporter expression in blue light. Reported fluorescence values in panels C-F are normalized to the optical density of the bacterial cultures and represent mean ± s.d. of three biologically independent samples, with the underlying individual measurements shown as white circles. Measurements under dark and light conditions were compared using a two-sided *t*-test with unequal variances; significance levels are shown above the bars and denote ***P < 0.001, **P < 0.01, *P < 0.05, n.s.: not significant. See Supplementary Figure S2 for predicted secondary structure, Supplementary Table S1 for sequences, and Supplementary Table S1 for calculated RNA stabilities of all circuit variants in panels C-F.

The earlier pCrepusculo-*Ds*Red plasmid (33), harboring a *Ds*Red Express 2 reporter gene (34), served as the template for constructing the initial riboptoregulator (RoR) variant RoR0. Using PCR amplification with overhang primers and Gibson assembly (35), the 5′-untranslated region upstream of the reporter gene was replaced by a *cis*-repressing RNA with the PAL motif-3 aptamer interleaved (see Figure 1B). Derivative riboptoregulator variants were generated by PCR amplification with overhang primers and blunt-end ligation. The pAurora2 plasmid was constructed by introducing the riboptoregulator circuit RoRH into the 5′-UTR of the *Ds*Red reporter gene within the pAurora-*Ds*Red vector (33) via PCR amplification and blunt-end ligation. The pREDusk-RoRH plasmid was generated in three steps. First, the PAL expression cassette was amplified from pCrepusculo and inserted into the pDusk-*Ds*Red vector (26) by Gibson assembly. Second, the riboptoregulator circuit RoRH was introduced into the 5′-UTR upstream of *Ds*Red by PCR amplification with overhang primers and blunt-end ligation. Finally, the LOV photosensor of the YF1 gene was replaced by a gene cassette comprising the *Synechocystis* sp. heme oxygenase 1 and the photosensory core module of the *Deinococcus radiodurans* bacteriophytochrome. To this end, the corresponding gene fragment was amplified by PCR from the pREDusk-*Ds*Red template (27) and subcloned by Gibson assembly. Empty versions of RoRH, RoRL, and pAurora2 were generated by PCR amplification with overhang primers and blunt-end ligation to introduce a multiple cloning site (MCS) that substitutes the *Ds*Red gene. These MCS plasmids will be made available through Addgene under accession numbers 213132, 213133 and 213134. For the bicistronic constructs, the gene encoding YPet was amplified from an earlier pCrepusculo-YPet plasmid (33) and cloned into RoRH-*Ds*Red by Gibson assembly and blunt-end ligation. A control construct constitutively expressing YPet was obtained by removing the PAL aptamer from the pCrepusculo-YPet plasmid via intramolecular Gibson assembly. The identity of all constructs was confirmed by Sanger DNA sequencing (Microsynth, Göttingen, Germany).

To develop riboptoregulator circuits with enhanced properties, the RoR0 and RoR12 variants were subjected to random mutagenesis. To this end, the 5′-UTR upstream of the *Ds*Red reporter gene, containing the RoR variant in question, was amplified by error-prone PCR using *Taq* polymerase with 5 mM MgCl_2_, 50 μM MnCl_2_, 0.8 mM dCTP and 0.8 mM dTTP added to the reaction mix (36). In parallel, the backbone of the vectors was amplified by conventional PCR using Phusion polymerase. The resulting PCR fragments from the two reactions were assembled (35) and transformed into *E. coli* CmpX13 cells (37). Upon plating on solid lysogeny broth (LB) agar supplemented with 100 μg mL^−1^ streptomycin (LB/Strep), the bacteria were incubated overnight at 37°C under constant illumination (470 nm, 40 μW cm^−2^). All light intensities were determined with a Newport (Darmstadt, Germany) 842-PE power meter and a Newport 918D-UV-OD3 silicon photodiode. Bacterial clones exhibiting higher fluorescence than the parental RoR constructs were streaked onto two LB/Strep agar plates which were incubated in darkness or under blue light (470 nm, 40 μW cm^−2^), respectively. Clones with pronounced differences in reporter fluorescence between the two illumination conditions were isolated, analyzed further, and identified by DNA sequencing.

### Reporter-gene assays

The response to blue light of the diverse riboptoregulator and pAurora variants was determined by monitoring the fluorescence of a *Ds*Red reporter and, in case of the bicistronic constructs, of a YPet reporter (33). *E. coli* CmpX13 clones carrying an optoribogenetic gene-expression circuit were used to inoculate 500 μl LB/Strep medium within individual wells of a 96-deep-well microtiter plate (MTP). The MTP was sealed and incubated for 16–18 h at 37°C while shaking at 600 rpm. Next, twice 2 μl of the cell suspensions were transferred to twice 198 μl LB/Strep medium within two separate transparent 96-well MTPs. The two plates were sealed with gas-permeable film (BF-410400-S, Corning) and incubated for 24 h at 37°C in darkness or under blue light (470 nm, 60 μW cm^−2^) while shaking at 800 rpm. For the analyses of the bicistronic operons, the cultures were instead illuminated from below at a wavelength of (463 ± 12) nm and an intensity of 60 μW cm^−2^ using a programmable 8 × 8 LED matrix (38). After incubation, the optical density at 600 nm (OD_600_) of the bacterial cultures was determined with a M200pro MTP reader (Tecan Group Ltd, Männedorf, Switzerland). Upon suitable dilution, the *Ds*Red fluorescence was recorded with the MTP reader using excitation and emission wavelengths of (554 ± 9) nm and (591 ± 20) nm, respectively. To measure YPet fluorescence, excitation and emission wavelengths of (500 ± 9) nm and (530 ± 20) nm, respectively, were used instead. The fluorescence readings were normalized by OD_600_ and are reported as mean ± s.d. of at least three biologically independent replicates. Two-sided *t*-tests with unequal variances were performed with Microsoft Excel.

The light-dose response of select optoribogenetic circuits was probed with a programmable eight-by-eight matrix of blue, green, and red LEDs emitting at (463 ± 12), (521 ± 14) and (624 ± 8) nm, respectively (33,38). To this end, *E. coli* CmpX13 cells harboring the circuit in question were cultured overnight at 37°C in 5 ml LB/Strep medium in darkness. The saturated starter culture was diluted 100-fold in fresh medium and dispensed in 200-μl aliquots into individual wells of a 96-well black-walled clear-bottom μClear plate (Greiner BioOne, Frickenhausen, Germany). Upon sealing with gas-permeable film, the MTP was placed on top of the LED array and incubated at 37°C for 24 h while shaking at 750 rpm. During incubation, each well was exposed to an individualized regime of blue (and, where applicable, red) light of variable intensity. Following incubation, the *Ds*Red reporter fluorescence of the cultures was measured, normalized, and averaged as described above. The variation of normalized reporter fluorescence *F* with blue-light intensity *I* was evaluated with Fit-o-mat (39) according to Equation (1):


(1) \begin{eqnarray*}F\left( t \right) = {F}_0 + {F}_1 \times I/\left( {I + {I}_{50}} \right)\end{eqnarray*}

where *I*_50_ denotes the light intensity at which the regulatory response manifests to half-maximal extent.

### Induction kinetics

To determine the kinetics with which gene expression ramps up upon blue-light exposure, triplicate starter cultures of *E. coli* CmpX13 bacteria containing the RoRH or RoRL circuits were grown overnight in 5 ml LB/Strep in darkness at 37°C and 225 rpm shaking. Then, 3 × 1 ml of the starter cultures were used to inoculate 3 × 100 ml LB/Strep within 250-ml baffled flasks, followed by incubation in darkness at 37°C and 225 rpm shaking. Once an *OD*_600_ of ∼ 0.5 was reached, the cultures were exposed to 60 μW cm^−2^ 470-nm light, and the cultivation continued. Aliquots were drawn at 0, 0.5, 1.5, 2.5, 3.5, 4.5, 5.5, 6.5, 7.5 and 24 h after the onset of blue light and were immediately arrested by addition of 0.4 mg ml^−1^ tetracycline and 3.5 mg ml^−1^ chloramphenicol (26). To allow complete maturation of *Ds*Red (34), samples were kept on ice for at least 2 h, followed by fluorescence measurements as above. To determine the time *t*_50_ of half-maximal gene expression, the *Ds*Red fluorescence normalized by *OD*_600_ was evaluated as a function of the time *t* at inducing blue-light conditions and fitted to a modified logistic function (26) using Fit-o-mat (39) (Equation 2).


(2) \begin{eqnarray*}F\left( t \right) = A + C \times {\left\{ {1 + \left( {{2}^T - 1} \right)\times {\mathrm{exp}}\left[ { - B \times \left( {t - {t}_{50}} \right)} \right]} \right\}}^{ - 1/T}\end{eqnarray*}

*A*, *B*, *C* and *T* are parameters that determine the position, amplitude, and steepness of the logistic function.

To assess the influence of blue light on bacterial growth and expression, triplicate bacterial starter cultures harboring either a pCDF-Duet empty vector or a positive-control plasmid that drives constitutive *Ds*Red expression were grown overnight at 37°C in darkness. Next, each triplicate culture was diluted 100-fold into 2 × 100 ml LB/Strep medium, followed by incubation at 37°C and 225 rpm shaking in darkness or 60 μW cm^−2^ 470-nm light. Aliquots were drawn at defined timepoints, arrested, and evaluated as described above.

### Flow cytometry

For single-cell fluorescence analyses, *E. coli* CmpX13 cultures carrying the RoRL, RoRH, and pAurora2 circuits, all configured with *Ds*Red reporter genes, were grown in MTPs in darkness or under constant blue light (470 nm, 60 μW cm^−2^) as described for the bacterial reporter-gene assays. An *E. coli* culture with the pCDF-Duet plasmid served as an empty-vector negative control. After incubation, the cultures were diluted 20- to 100-fold in phosphate-buffered saline (1 × sheath fluid, BioRad) and analyzed on an S3e cell sorter (BioRad) equipped with 488-nm and 561-nm excitation lasers. For each culture, around 200 000 cells were analyzed, and their single-cell fluorescence [emission at (585 ± 15) nm] was evaluated. Data processing was performed with custom Python scripts (available at https://github.com/TheAngulion/fcs2txt). The binned single-cell fluorescence distribution was fitted to log-normal distributions using Fit-o-mat (39). All experiments were conducted for at least three biologically independent replicates and yielded consistent results.

## Results

### Riboptoregulator design

To explore novel modalities for optoribogenetic control, we derivatized the earlier pCrepusculo-*Ds*Red plasmid (33) which affords blue-light-repressed bacterial gene expression. Notably, the plasmid encodes the *Nm*PAL gene under control of a constitutive promoter and a *Ds*Red reporter gene which enables ready expression analyses via fluorescence measurements. As noted above, expression of the reporter is subjected to blue-light control by interweaving an RNA aptamer, denoted motif 3 (33), with the Shine-Dalgarno sequence that is situated within the 5′-UTR where it constitutes part of the ribosome-binding site. Triggered by blue light (470 nm), *Nm*PAL binds the motif-3 aptamer and sequesters the SD in a base-paired RNA stem, thereby causing impaired ribosome binding and 19-fold lower reporter expression compared to darkness (Supplementary Figure S1).

Inspired by the seminal riboregulator design (12), we reprogrammed the pCrepusculo circuit by introducing into the 5′-UTR of the *Ds*Red reporter gene a *cis*-repressing RNA (*cis*-RNA) stretch that can adopt an extended stem-loop structure which masks the SD sequence and thus restricts translation initiation (Figure 1A).

To install sensitivity to light, we introduced the motif-3 aptamer into this stem loop such that the *cis*-RNA can assume two mutually exclusive base-paired states (Figure 1A, B, Supplementary Table S1). In a closed state, the *cis*-RNA folds into the extended stem loop that restricts access to the SD region. Alternatively, an open state results if the motif-3 aptamer folds into a shorter stem loop which concomitantly disrupts base-pairing in other parts of the *cis*-RNA and thereby grants access to the SD region. Given the lower extent of base pairing within the open state, the conformational equilibrium is intrinsically tilted towards the closed state (Supplementary Table S2). Once activated by blue light, *Nm*PAL would bind to the motif-3 aptamer in its folded hairpin structure and thus shift the equilibrium to the open state, relieve translational repression, and allow gene expression to ensue (Figure 1A).

To put this design rationale to the test, we devised a first circuit variant with several mismatches, internal loops, and bulge loops (termed MM, IL, and BL, respectively) that deliberately introduce base-pairing defects into the *cis*-RNA and that should therefore stabilize the open at the cost of the closed state (Figure 1B, Supplementary Tables S1 and S2). *E. coli* bacteria harboring this circuit and cultured overnight at 37°C exhibited around 7-fold higher reporter fluorescence under 60 μW cm^−2^ blue light (470 nm) than in darkness (Figure 1C), thereby validating the fundamental design hypothesis. Given the pronounced light response, we dubbed the initial circuit riboptoregulator (RoR) 0.

We next set out to probe and optimize RoR performance by both rational and random approaches. In a first construct series, we individually closed the internal and bulge loops within the *cis*-RNA and assessed the effects on light-dependent reporter fluorescence. Whereas the removal of a mismatch between the SD region and the start codon (MM1) had little effect (Figure 1B, D, Supplementary Figure S2A, Supplementary Tables S1 and S2), closure of IL1 and BL1, both situated within the SD region, lowered the reporter fluorescence, arguably due to the resultant stabilization of the closed *cis*-RNA state. As, in the case of BL1, this effect was more pronounced in darkness, the expression difference between blue light and darkness, referred to as the dynamic range of regulation in the following, increased to around 15-fold. Removal of base mismatches in the *cis*-RNA within the region of the motif-3 aptamer had little or even adverse effect on the dynamic range (variants BL2 and IL2 in Figure 1D). By contrast, closure of IL3, at the junction between the SD sequence and the motif-3 aptamer elevated the reporter expression under blue light and thereby increased dynamic range to 13-fold.

Next, we evaluated the effect of modifying the size of the motif-3 aptamer while maintaining the total length of the *cis*-RNA (Figure 1E, Supplementary Figure S2B). For RoR0, this aptamer comprises 18 nucleotides (nt) that can fold into a stem loop. Shortening of the stem region in two-nt increments gave rise to aptamers of 14 and 16 nt size, which exhibited reduced gene expression under light compared to RoR0 and accordingly worse dynamic range. By contrast, extending the aptamer to a size of 20 nt resulted in higher reporter fluorescence than for RoR0 but lower dynamic range. Although the variation of the aptamer size did not improve RoR performance, the findings are consistent with the underlying design rationale. Stabilization of the aptamer by extending its stem size is fully expected to favor the open *cis*-RNA state and thus promote higher gene expression.

Lastly, we varied the distance between the motif-3 and SD regions within the *cis*-RNA by successively removing base pairs from the intervening junction (Figure 1F, Supplementary Figure S2C). Unexpectedly, deletion of 1 base pair (bp) abolished the light response and yielded constitutively high reporter fluorescence. Retrospective analysis revealed that the deletion inadvertently resulted in a motif-3 aptamer with an extended stem and a total size of 28 nt which strongly and largely independently of light shifted the conformational balance to the open state of the *cis*-RNA. By contrast, removal of two successive bp had little impact on the light response compared to RoR0. Shortening by 3 or 4 bp substantially reduced the reporter fluorescence both in darkness and under blue light. The variant with a 3-bp removal, denoted RoR12 subsequently, exhibited an improved, 14-fold dynamic range of light regulation.

**Figure 2.**
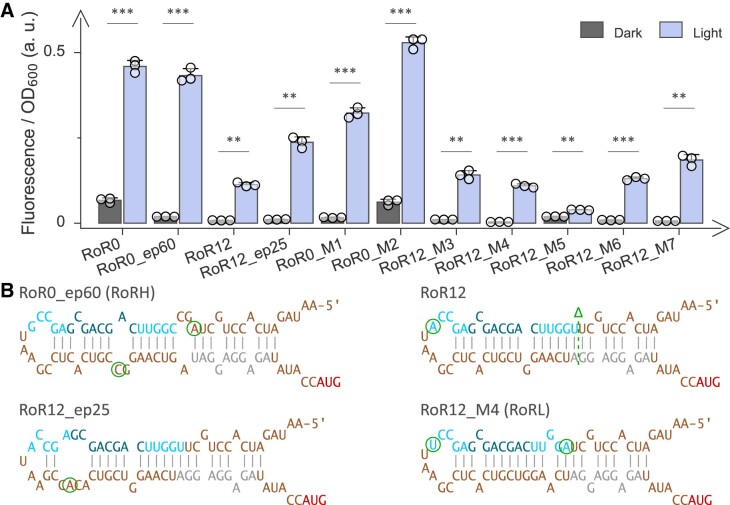
Optimization of the RoR circuit by rational and random modifications. (**A**) Menagerie of RoR circuits and their normalized *Ds*Red reporter fluorescence in darkness (grey bars) and under blue light (blue bars), respectively. Starting from RoR0, the RoR0_ep60 variant (termed RoRH) arose from error-prone PCR (epPCR). Likewise, RoR12_ep25 originates from random mutagenesis of the RoR12 circuit (cp. Figure 1E). Modifications within the two variants identified by epPCR were iteratively introduced into the parental circuits RoR0 and RoR12, respectively (see Supplementary Figure S3). Doing so yielded the variant RoR12_M4, named RoRL, with low basal expression and stringent light regulation. Reported fluorescence readings are mean ± s.d. of three biologically independent samples, with the underlying individual measurements shown as white circles. Measurements under dark and light conditions were compared using a two-sided *t*-test with unequal variances; significance levels are shown above the bars and denote ***P < 0.001, **P < 0.01, *P < 0.05, n.s.: not significant. (**B**) Predicted secondary structure (40) of select circuit variants from panel A. For the other variants, see Supplementary Figure S3.

We complemented the rational RoR variation by random mutagenesis using error-prone PCR and Gibson assembly (Figure 2). Following mutagenesis restricted to the *cis*-RNA region, the resulting construct libraries were screened for variants displaying stronger reporter fluorescence under blue light than the starting template. Separate mutagenesis campaigns were carried out for RoR0 and RoR12. This way, we identified two new clones called RoR0_ep60 and RoR12_ep25 deriving from the indicated parental circuits that exhibited dynamic ranges of light regulation of 22-fold and 21-fold, respectively (Figure 2A-B, Supplementary Table S1-S2). Given its superior light response, we refer to the RoR0_ep60 variant as RoRH. Sequencing revealed that RoRH harbors two mutations compared to RoR0, one each within the SD and aptamer regions of the *cis*-RNA. Individual analyses of the two mutations, constructs RoR0_M1 and RoR0_M2 in Figure 2A, largely pinpointed the beneficial effect to a nucleotide exchange that converted a G:U to an A:U base pair within the SD region (Figure 2A, Supplementary Figure S3A). As a corollary, in the RoR0_M1 construct basal expression in darkness reduced, and the dynamic range amounted to > 20-fold. The RoR12_ep25 variant bore one mutation that diminished the extent of base pairing within the aptamer region in the closed *cis*-RNA state.

We next applied the residue exchanges identified by random mutagenesis in the RoR0 background to the RoR12 context (Figure 2A, B, Supplementary Figure S3B, Supplementary Tables S1 and S2). Most interestingly, introduction of the same exchange as in RoR0_M1 plus a compensatory mutation to maintain aptamer size yielded the RoR12_M4 circuit with exceedingly low basal expression in darkness and a 30-fold dynamic range of light regulation. Given its performance, we renamed this variant as RoRL and analyzed it further alongside the above RoRH.

### Riboptoregulator characterization

**Figure 3.**
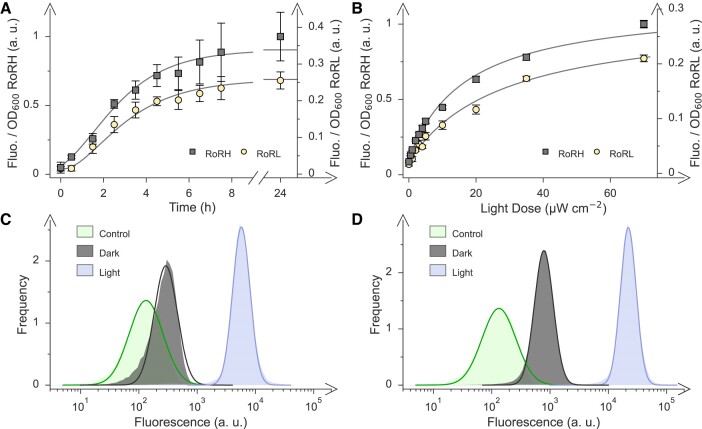
Light-dependent regulation of bacterial expression by the riboptoregulators RoRL and RoRH. (**A**) Induction kinetics of the RoR circuits. Upon exposure to blue light (470 nm, starting at time zero), the *Ds*Red reporter-gene expression, normalized to the optical density of the bacterial cultures, ramped up with times *t*_50_ of half-maximal expression of (2.9 ± 0.3) h for RoRL (yellow circles) and (2.3 ± 0.9) h for RoRH (grey squares). (**B**) Light-dose response of RoRL (yellow circles) and RoRH (grey squares). The half-maximal light doses *I*_50_ amounted to (28 ± 6) μW cm^−2^ for RoRL and (17 ± 2) μW cm^−2^ for RoRH. The reported fluorescence readings in panels A and B correspond to mean ± s.d. of three biologically independent replicates. (**C**) Single-cell fluorescence of bacteria harboring the RoRL circuit and incubated in darkness (grey) or blue light (blue), with an empty-vector control displayed in cyan. The solid lines denote fits to log-normal distributions and yielded median fluorescence values of 10^2.5^ arbitrary units (a.u.) in darkness and 10^3.8^ a.u. under blue light, compared to 10^2.1^ a.u. for the empty-vector control. (**D**) As panel C but for RoRH. The median fluorescence values in darkness and blue light amounted to 10^2.9^ and 10^4.3^ a.u., respectively. Data in panels C and D are based on the analyses of at least 200 000 individual cells each.

The iterative optimization of the riboptoregulator circuit pinpointed the variants RoRL and RoRH with low and high maximal expression levels, respectively, and both with dynamic ranges of light regulation exceeding 20-fold. To facilitate optoribogenetic deployment, we assessed the response to light of these circuits in more detail. First, we investigated the induction kinetics upon exposure to blue light. To this end, bacteria harboring either circuit were cultured in darkness before transfer to continuous blue light (470 nm, 60 μW cm^−2^). Aliquots were drawn at different times afterwards, translationally and transcriptionally arrested, and further incubated to allow for *Ds*Red maturation (Figure 3A). For both RoRL and RoRH, the reporter fluorescence per cell count increased sigmoidally with times *t*_50_ of half-maximal activation of (2.9 ± 0.3) h and (2.3 ± 0.9), respectively, similar to the induction kinetics recorded for pCrepusculo upon transfer from blue light to darkness (33).

Second, we examined the dose-response relationships of RoRL and RoRH by cultivating bacteria carrying these circuits under different blue-light intensities. The reporter fluorescence increased hyperbolically with half-maximal light intensities *I*_50_ of (28 ± 6) and (17 ± 2) μW cm^−2^ for RoRL and RoRH, respectively (Figure 3B). Interestingly, these *I*_50_ parameters are way above the value of around 1 μW cm^−2^ for the pCrepusculo setup from which the riboptoregulator circuits derive (33). Notably, both the riboptoregulator and the pCrepusculo circuits harness the same motif-3 aptamer, and they employ the same constitutive promoter to drive *Nm*PAL expression. Although a full molecular understanding is currently lacking, we speculate that the divergent light sensitivities may be rooted in the different RNA contexts the motif-3 aptamer is embedded in. Whereas in pCrepusculo, the *Nm*PAL receptor modulates as a function of blue light the balance between short-hairpin and unfolded RNA structures, in the riboptoregulator it is an equilibrium between two folded RNA structures, i.e. the open and closed states of the *cis*-RNA, that is subject to regulation. Integration into the *cis*-RNA may well cause a lower affinity of the motif-3 aptamer for *Nm*PAL. Consequently, more *Nm*PAL molecules would need to be activated by light which would translate into a lower light sensitivity, as indeed observed experimentally. Irrespective of the precise molecular mechanism at play, the current experiments illustrate that both RoRL and RoRH follow conventional dose-response behaviors and can be toggled at moderate blue-light intensities in the range of a few ten μW cm^−2^.

To assess whether blue light impacts on bacterial growth, we inspected the density of the bacterial cultures for the above illumination regimes (Supplementary Figure S4A, B). Notably, these cultures attained cell densities upon cultivation which were essentially independent of the applied light dose, thus indicating that at the presently used intensities (up to 60 μW cm^−2^), blue light does not significantly impair bacterial proliferation. In a similar vein, bacteria harboring either an empty vector or a plasmid driving constitutive *Ds*Red expression exhibited closely similar growth kinetics in darkness and under 60 μW cm^−2^ blue light (Supplementary Figure S4C, D). Likewise, extent and kinetics of the constitutive *Ds*Red expression were unaffected by illumination (Supplementary Figure S4E).

Beyond the above ensemble measurements, we also analyzed the RoRL and RoRH circuits at the single-cell level to assess the homogeneity of the population and its response to light exposure (Figure 3C, D). Using flow cytometry, we observed homogenous log-normal frequency distributions of single-cell fluorescence for both circuits and under both dark and blue-light conditions (470 nm, 60 μW cm^−2^). In the case of RoRL, the median fluorescence values of the distributions in darkness and blue light amounted to 10^2.5^ and 10^3.8^ arbitrary units (a.u.), respectively, corresponding to a 20-fold difference. For reference, the median fluorescence of control bacteria carrying an empty vector without fluorescent reporter was 10^2.1^ a.u., i.e. 2.5-fold lower than the median value for RoRL in darkness. For RoRH, we determined median fluorescence values in darkness and blue light of 10^2.9^ and 10^4.3^ a.u., respectively, reflecting a 25-fold shift. Taken together, both RoR circuits respond to illumination stringently and essentially in an all-or-none manner; no subpopulations of unresponsive cells were detected. As the observed shifts of the median positions exceed the widths of the frequency distributions, there is no overlap in single-cell fluorescence between dark and blue-light conditions.

### Integrated circuits for enhanced optogenetic control

While the RoRL and RoRH circuits stringently activate bacterial expression under blue light, the dynamic range of regulation they attain pales in comparison to certain transcription-based optogenetic circuits which often achieve induction by several hundred-fold (19,24,26–28). At the same time, the riboptoregulator circuits compare favorably to alternative means of regulating bacterial expression at the level of translation, for instance, via riboswitches (10,41,42), that frequently exhibit single-digit regulatory responses to signal only.

**Figure 4.**
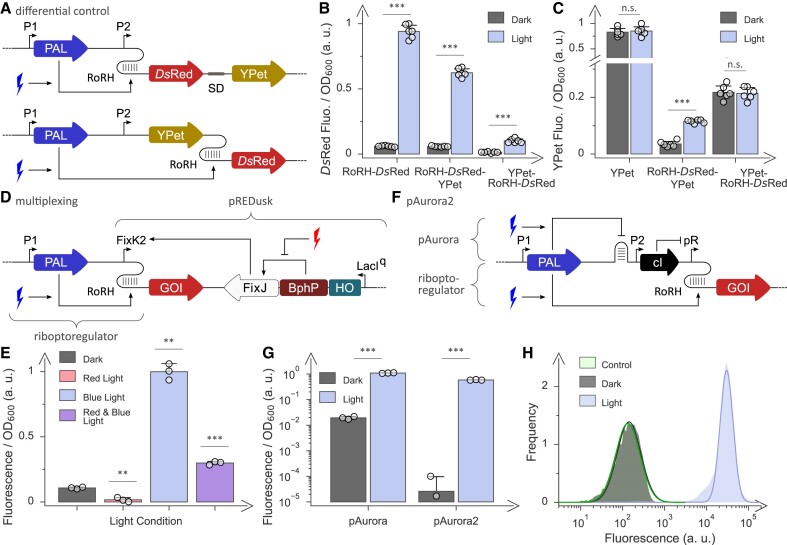
Integrated and multiplexed optogenetic control. (**A**) The riboptoregulator (RoR) circuit responds to blue light and operates at the mRNA level, thus lending itself to the differential control of bicistronic operons. Two operon variants differ in the sequential order of the YPet and *Ds*Red fluorescence reporters, with the latter reporter under control of the RoRH circuit. (**B**) *Ds*Red reporter fluorescence of the circuits depicted in panel A following incubation in darkness (grey bars) or blue light (blue). The monocistronic RoRH-*Ds*Red is shown for comparison. (**C**) YPet reporter fluorescence of the circuits from panel A. A construct constitutively expressing YPet is shown for comparison. (**D**) The riboptoregulator circuit RoRH responds to blue light and operates at the mRNA level, thus rendering it orthogonal to the pREDusk circuit (27) which is sensitive to red light and controls transcription initiation. The two circuits can hence be integrated to jointly control the expression of a gene of interest (GOI). (**E**) Response of the multiplexed pREDusk-RoRH setup depicted in panel D. *E. coli* bacteria harboring this setup were incubated in darkness (grey bar), under blue light (blue bar), under red light (red bar) or under both blue and red light (purple bar). (**F**) The earlier pAurora circuit (33), based on *Nm*PAL-mediated repression of the λ phage cI repressor, was upgraded by integration with the riboptoregulator RoRH. Within the resultant pAurora2 system, expression of the gene of interest is thus controlled at both the transcriptional level (via the cI repressor) and the translational level (via *Nm*PAL). (**G**) Reporter fluorescence of bacteria carrying the pAurora or pAurora2 plasmids following incubation in darkness or under blue light. The fluorescence values reported in panels B, C, E and G are normalized to the optical density of the bacterial cultures and reflect mean ± s.d. of at least three biologically independent replicates (individual measurements shown as white circles). Measurements under different illumination conditions were compared to the dark condition using a two-sided *t*-test with unequal variances; significance levels are shown above the bars and denote ***P < 0.001, **P < 0.01, *P < 0.05, n.s.: not significant. (**H**) Single-cell fluorescence distribution of bacteria harboring pAurora2. When incubated in darkness (grey) or under blue light (blue light), the median fluorescence was 10^2.15^ arbitrary units (a.u.) and 10^4.5^ a.u., respectively, which compares to 10^2.12^ a.u. for the empty-vector control (cyan). For each data set, at least 200 000 single cells were analyzed.

A key advantage of the riboptoregulator strategy is its action at the mRNA level. We reasoned that this aspect might allow the differential control of individual genes within polycistronic operons that are the norm in bacteria. To test this concept, we devised bicistronic operons that combine the YPet and *Ds*Red fluorescence reporters, with the latter being under RoRH control (Figure 4A). Two circuit variants differing in the sequential order of the YPet and RoRH-*Ds*Red cassettes showed 7- to 11-fold higher *Ds*Red expression under blue light compared to darkness, a slightly weaker but overall similar response as in the isolated, monocistronic RoRH-*Ds*Red under these conditions (Figure 4B). By contrast, the YPet expression was induced only 3-fold or not at all, depending on whether the YPet gene was placed downstream or upstream of the RoRH-*Ds*Red cassette (Figure 4C). Collectively, the data illustrate how the riboptoregulator can be used to individually address specific members within an operon.

Another advantage of expression control at the mRNA level is that transcription is bypassed, and downstream effects may manifest faster than for transcription-based circuits. Moreover, circuits acting at the RNA level, such as pCrepusculo and the riboptoregulator, lend themselves to combinations with setups that target the DNA level. Capitalizing on this principle, we combined riboptoregulator circuits with existing setups for optogenetic expression control to arrive at integrated circuits with emergent and improved regulatory traits.

We thus expanded the recent pREDusk system that downregulates bacterial expression in response to red light (27) (Figure 4D). Briefly, this system comprises a chimeric bacteriophytochrome histidine kinase (BphP) that in concert with a response regulator (RR) controls transcription of genes of interest (GOI). We installed the RoRH module within the 5′-UTR of the GOI (i.e. *Ds*Red) and placed the gene cassette for constitutive *Nm*PAL expression onto the same plasmid. Next, we probed the response of bacteria harboring the resulting integrated plasmid, termed pREDusk-RoRH, to blue and red light (Figure 4E). The low reporter fluorescence in darkness could be dialed up by 9-fold or down by 7-fold under blue and red light, respectively, whereas exposure to both light colors prompted a 3-fold fluorescence increase. Evidently, the composite pREDusk-RoRH circuit responded to and integrated dual light inputs into defined expression output, thus granting finer-grained optogenetic control. More broadly, the circuit exemplifies the construction of integrated circuits that heed and process several light qualities which not least stands to be relevant for applications in strictly photoauxotrophic organisms that require light for survival (43).

As illustrated by the above example, circuit integration can benefit (optogenetic) regulation. The fundamental concept also holds true for combinations of circuits responding to the same light color which may be assembled to achieve ever-more stringent optogenetic expression control. This notion finds support in an elegant study that combined blue-light-sensitive circuits controlling target protein levels at the transcriptional, translational, and posttranslational stages to achieve regulatory dynamic ranges much exceeding those possible for the circuits deployed separately (44). In the arguably simplest case, the individual subcircuits of an integrated system even share the same photoreceptor entity, thus making for a compact architecture.

We tested this concept by inserting the RoRH circuit upstream of the *Ds*Red reporter gene within the previous pAurora-*Ds*Red plasmid (33) (Figure 4F). In the upgraded system, named pAurora2, light-induced *Nm*PAL binding to its aptamer activates reporter gene expression via two routes. Within the riboptoregulator branch of the circuit, aptamer binding relieves translational repression of the reporter and thereby ramps up its expression. A second branch, corresponding to pAurora (33), harnesses aptamer binding to translationally repress the expression of the λ phage cI repressor which in turn negatively regulates the promoter controlling reporter expression. pAurora2 exhibited outstanding performance with minimal reporter fluorescence in darkness and > 1000-fold upregulation under blue light (Figure 4G). As the fluorescence signal in darkness is at the level of the background caused by bacterial autofluorescence, as assessed with an empty-vector control, a more precise quantification is hampered.

Not least to overcome this challenge, we analyzed the response of bacteria carrying pAurora2 to blue light at the single-cell level (Figure 4H). Upon cultivation in darkness, the bacteria displayed uniform single-cell fluorescence distributed around a median value of 10^2.15^ a.u. which is very close to the background fluorescence of the empty-vector control centered at 10^2.12^ a.u. Blue light prompted a shift of the median fluorescence to 10^4.5^ a.u. which was largely uniform with a minor tail at slightly lower fluorescence values. Taken together, these data compellingly underline the extraordinarily stringent optogenetic response afforded by the pAurora2 circuit. By contrast, as separate systems, the parental pAurora and RoRH circuits achieved much lower dynamic ranges of light regulation of around 70- and 25-fold, respectively. More generally, circuit integration can bring about synergy and thereby yield traits that transcend the individual subcircuits. If the constitutive subcircuits are mutually orthogonal, e.g. because they operate at different stages of the gene-expression trajectory, integration can become as straightforward as it is efficient.

## Discussion

Our study adds to the growing arsenal of optoribogenetic circuits for light control at the RNA level (29–33,45). At present, we leverage RNA refolding transitions to channel light-activated RNA binding by the LOV receptor *Nm*PAL into the desired output, i.e. the activation of bacterial gene expression. Doing so provides ample opportunities for further fine-tuning, for instance by varying the length and melting temperature of the *cis*-RNA, as demonstrated for the original, light-inert riboregulator setup (12). The riboptoregulator concept should principally extend to other RNA-based circuits and bestow light sensitivity onto them. Of particular interest, the so-called toehold switches are a riboregulator class with modified base-pairing logic that achieve higher regulatory efficiency (46). Future efforts will be directed at interrogating whether the toehold switches can be also upgraded to riboptoregulators. LicV, an engineered, light-activated RNA-binding receptor (45), might also be used in similar capacity as presently realized and envisioned, thereby paving the way towards multiplexing of several regulatory circuits in parallel. However, compared to *Nm*PAL, LicV relies on light-induced homodimerization (which incurs strong dependence on cellular expression levels (4)), binds its RNA target with weaker affinity, and exhibits a less pronounced dynamic range of light regulation.

Regulation at the RNA level, optoribogenetic (29) or otherwise (12,46), offers several benefits. First, pertinent approaches allow the differential control of gene products within polycistronic operons which prevail in prokaryotes (see Figure 4A–C). Enabled by the riboptoregulator strategy, individual structural genes of operons may now be upregulated by light cues which for instance stands to benefit the engineering and optimization of metabolic pathways (47,48). Second, regulation at the translational stage is expected to manifest in expression differences faster than regulation at the transcription level. Third, RNA-based regulatory circuits are often orthogonal to transcription-based regulatory circuits which facilitates circuit integration. As presently demonstrated (see Figure 4D–H), integration affords nuanced and more stringent control of gene expression than possible for a single circuit alone. Integration principally extends to numerous regulatory circuits that predominantly gate transcription initiation and that can hence be upgraded by the riboptoregulator.

These benefits may come to bear for modern applications of light-regulated bacterial gene expression in theranostics, biotechnology, and materials science (4). As diverse as the pertinent scenarios are, they generally bank on the capacity to activate and suspend expression with precision in time and space. In advancing pAurora2 which supports highly stringent expression responses to blue light, we provide a potent implement for exactly these use cases. Chiefly owing to miniscule basal expression in darkness, pAurora2 outperforms pDawn (26) and other widely used systems for the optogenetic control of bacterial expression (4,49). Given its highly stringent regulation combined with very low background activity, pAurora2 may not least become a promising option for the expression of toxic genes.

The riboptoregulator strategy devised at present generally applies to the deliberate tuning of bacterial expression. Put simply, we install light responsiveness in the riboregulators (12,46,50) which even in their original, light-inert forms see wide use in basic and applied research (51). Arguably, the broad application of riboregulators can be attributed to the specific advantages they afford (12,46,50–52), among them their inherent modularity which facilitates the embedding into diverse RNA contexts as further exemplified below; their comparatively small genetic footprint which reduces the metabolic burden; their ready programmability to target diverse RNA sequences; and the tunability of their output in response to input triggers. As but one example, a recent study explored the regulation of bacterial gene expression at the transcriptional and translational stages by various riboregulators (52). Irrespective of their diversity, conventional riboregulators generally rely on strand-displacement reactions (12,46,53) and use *trans*-activating RNAs (*ta*RNA) as input triggers. By contrast, the riboptoregulator method now substitutes the *ta*RNAs as the triggers for the light-activated binding of *Nm*PAL to its cognate RNA aptamer.

*Nm*PAL binding induces conformational changes in target RNAs which can promote the exposure or masking of sequence epitopes to thereby control downstream responses. This principal concept of modulating access to RNA epitopes in dependence of light extends to other scenarios. For instance, recent work showcased the application of riboregulators to grant or withhold access to guide RNAs (gRNA) of Cas12 endonucleases (54). Informed by our present work, riboptoregulators may be appended to the 5′ end of such gRNAs in similar fashion and thereby subject the catalytic activity of Cas12 and, prospectively, other programmable endonucleases to light control. In this manner, the endonucleolytic activity may be confined in time and undesired, excessive activity be curtailed (55).

In essence, the riboptoregulator may be regarded as an engineered riboswitch with an integrated aptamer domain that preferentially binds the light-adapted state of *Nm*PAL. Conventional riboswitches with aptamer domains sensitive to small molecules have proven equally versatile and proficient at regulating gene expression across prokaryotic and eukaryotic hosts. As a case in point, multiple studies employed theophylline and tetracycline riboswitches to control gene expression in both prokaryotic and eukaryotic cells (56–61). Given their mechanistic similarity to these synthetic riboswitches, riboptoregulator circuits might be used in a similar capacity, e.g. for controlling pre-mRNA splicing in mammalian cells (56–58) by blue light.

More broadly, the recent years have witnessed the emergence and vigorous refinement of diverse riboswitches, riboregulators, and other RNA-based circuits that can be now applied for gene regulation, synthetic biology, biomaterials, and synthetic cells, among other use cases (51,62). Irrespective of these advances, a pronounced dearth of light-sensitive circuits remains although the benefits and great potential of regulation by genetically encodable RNA photoswitches have been long recognized (63). Of note, RNA aptamers have been raised against photochromic ligands that preferentially bind one specific of several (meta) stable ligand conformations (64–67). For instance, a recent report (65) employed SELEX (68) to identify an RNA aptamer that selectively binds the *trans* isomer of an azobenzene compound but not the *cis* form. Although this and related aptamers lend themselves to the construction of light-dependent riboswitches, they are not fully genetically encoded as the photochromic ligand needs to be added exogenously. The departure from genetic encoding may severely restrict any application of these strategies in cells and *in vivo*.

Our present investigation addresses this unmet demand by illustrating how the *Nm*PAL:aptamer interaction can bestow light sensitivity on RNA circuitry while maintaining full genetic encoding. Akin to conventional riboregulators (53), the desired regulatory effect hinges on RNA conformational transitions. The iterative construction and testing of riboptoregulator variants (see Figures 1 and 2) conducted at present inform the future adaptation of the fundamental design strategy to other RNA molecules and circuits. Key to pronounced regulation by light is the relative stability of two competing RNA folds which can be modulated by both rational and random sequence variation.

In common with other optogenetic technologies (13,69), the riboptoregulator strategy provides tangible benefits over concurrent means of exerting cellular control. Owing to true genetic encoding and the use of light as a trigger, optogenetics enables the non-invasive, reversible, and spatiotemporally precise probing of cellular processes. Prospectively, these advantages may be tapped for the analyses of bacterial physiology, particularly when acuity in space and time is demanded (4). Relevant candidate scenarios include the control and study of cell-cell communication and dynamics within bacterial biofilms (70,71). Likewise, optogenetics may serve to modulate the interplay of microbial cell communities (72).

In closing, we note that light-inducible gene expression represents the predominant modality in bacterial optogenetics (4). However, the vast majority of available optogenetic methods target transcription initiation, e.g. (17,23,24,26–28,49,73). By contrast, the riboptoregulator approach stands apart by acting at the mRNA level and thereby timely expands the optogenetic repertoire by a hitherto unavailable, adaptable, and efficient tool.

## Supplementary Material

gkae678_Supplemental_File

## Data Availability

Primary data underlying the main and supplementary figures are provided at https://doi.org/10.5281/zenodo.11366027. The RoRL, RoRH, and pAurora2 plasmids will be distributed via Addgene under accession numbers 213132, 213133, and 213134. Custom Python code for processing and binning flow-cytometry data is available at https://github.com/TheAngulion/fcs2txt and https://zenodo.org/doi/10.5281/zenodo.12793994.
