# Supplementary material for: Induction of bacterial expression at the mRNA level by light
Source: Nucleic Acids Res. 2024 Aug 10;52(16):10017–28. doi: 10.1093/nar/gkae678 (PMC11381354; doi:10.1093/nar/gkae678)
Supplement: gkae678_Supplemental_File [file gkae678_supplemental_file.pdf]

Induction of Bacterial Expression at the mRNA Level by Light

Américo T. Ranzani[1,*], Konrad Buchholz[1], Marius Blackholm[1], Hayat Kopkin[1], Andreas Möglich[1,2,3,*]

[1] Department of Biochemistry, University of Bayreuth, 95447 Bayreuth, Germany

[2] Bayreuth Center for Biochemistry & Molecular Biology, Universität Bayreuth, 95447 Bayreuth, Germany

[3] North-Bavarian NMR Center, Universität Bayreuth, 95447 Bayreuth, Germany

* To whom correspondence should be addressed. Tel: +49-921-55-7835; Email: americo.ranzani@uni-bayreuth.de or andreas.moeglich@uni-bayreuth.de

Table of Contents

Supplementary Figure 1 ____ 2

Supplementary Figure 2 ____ 3

Supplementary Figure 3 ____ 4

Supplementary Figure 4 ____ 5

Supplementary Table 1 – Sequences of Riboptoregulator Circuits ____ 7

Supplementary Table 2 – Predicted Free Energy of RNA Structures within Riboptoregulator Circuits ____ 9

Supplementary References ____ 10

Supplementary Figure 1

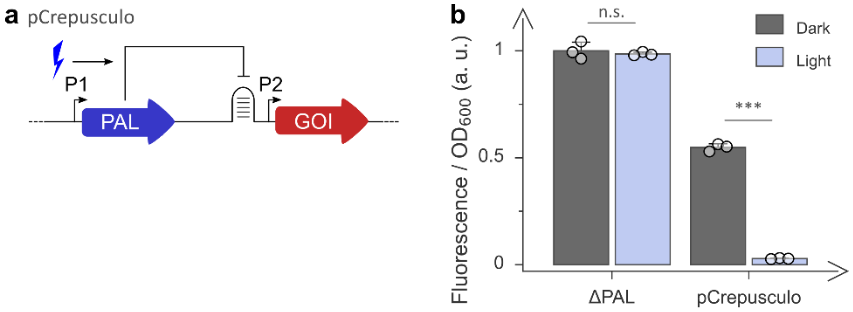

The pCrepusculo (1) circuit for regulation of bacterial expression at the mRNA level. **a**, Circuit schematic. Expressed from a constitutive promoter P1, *Nm*PAL can be activated by blue light. Subsequent binding to a specific aptamer that overlaps with the Shine-Dalgarno sequence of a gene of interest (GOI) incurs a reduction in expression of said gene. **b**, Normalized *Ds*Red reporter fluorescence of bacteria harboring the pCrepusculo plasmid when incubated in darkness (grey bars) and under blue light (blue bars), respectively. The illumination of the culture caused a 19-fold decrease in fluorescence. A control plasmid (ΔPAL) harbors no *Nm*PAL. The fluorescence values are normalized to the optical density of the bacterial cultures and reflect mean ± s.d. of three biologically independent replicates, with individual measurements shown as white circles. Measurements under dark and light conditions were compared using a two-sided t-test with unequal variances; significance levels are shown above the bars and denote ***: $p < 0.001$, n.s.: not significant.

Supplementary Figure 2

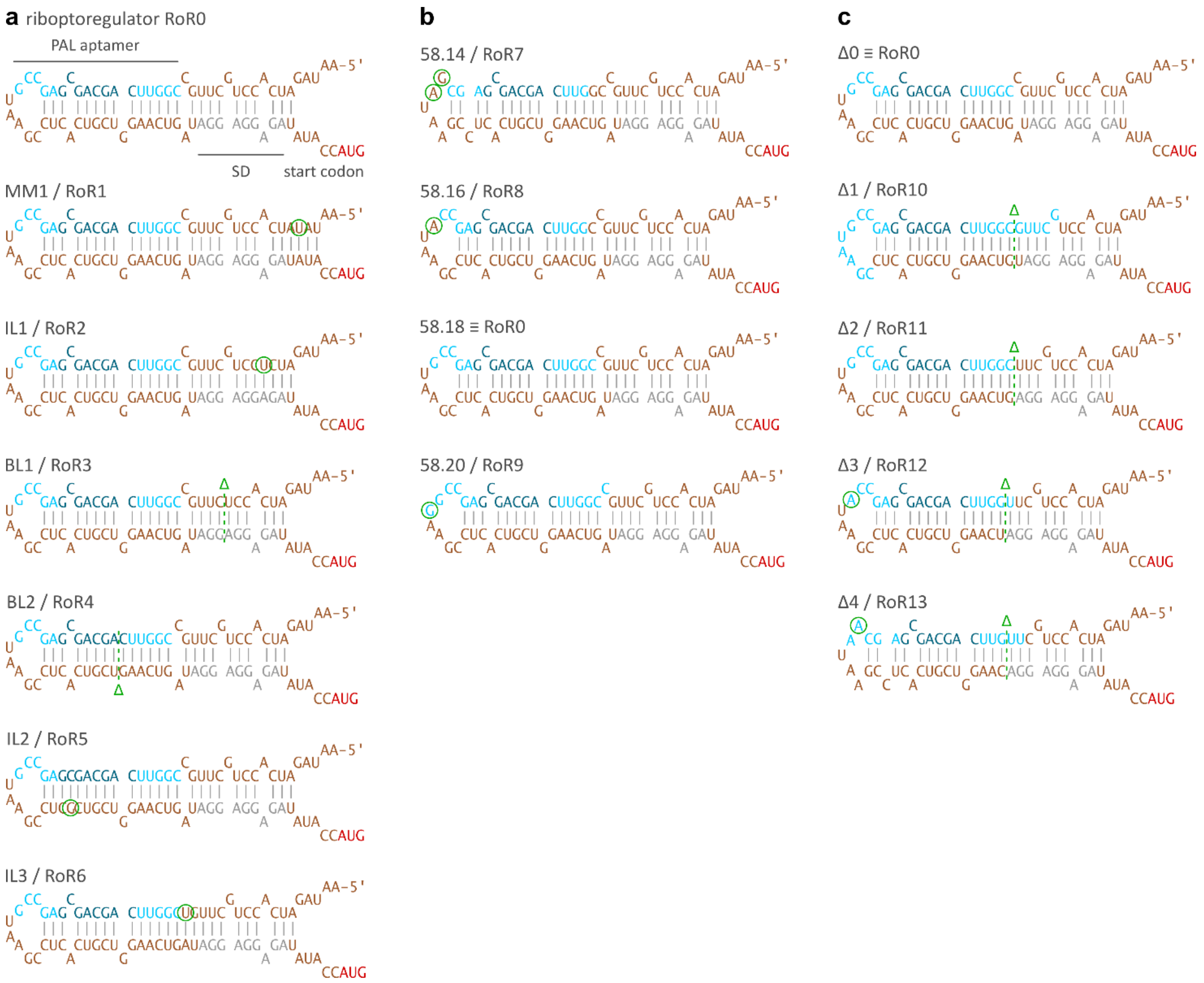

Predicted secondary structure of the riboptoregulator circuit variants shown in Fig. 1. The original, starting design is denoted RoR0, and changes relative to that design are highlighted in green. **a**, Closure of unpaired regions (see Fig. 1d). **b**, Variation of the PAL aptamer size (see Fig. 1e). The original RoR0 is based on the motif-3 aptamer (1) with a size of 18 bases that is denoted as 58.18. **c**, Variation of the overlap between PAL aptamer and Shine-Dalgarno sequence relative to the original RoR (designated Δ0) (see Fig. 1f). All secondary structure predictions were done with RNAfold (2).

Supplementary Figure 3

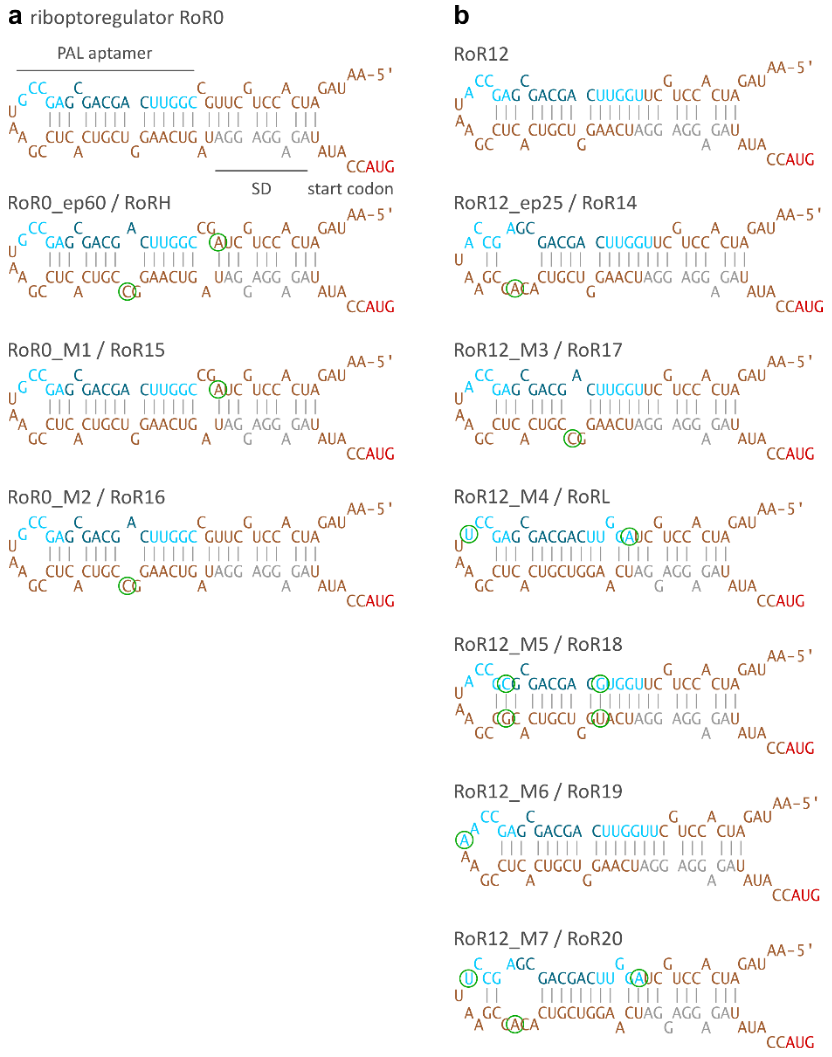

Predicted secondary structure (2) of the riboptoregulator circuit variants shown in Fig. 2. The original, starting design is denoted RoR0, and changes relative to that design are highlighted in green. **a**, Derivatization of RoR0. **b**, Derivatization of RoR12.

Supplementary Figure 4

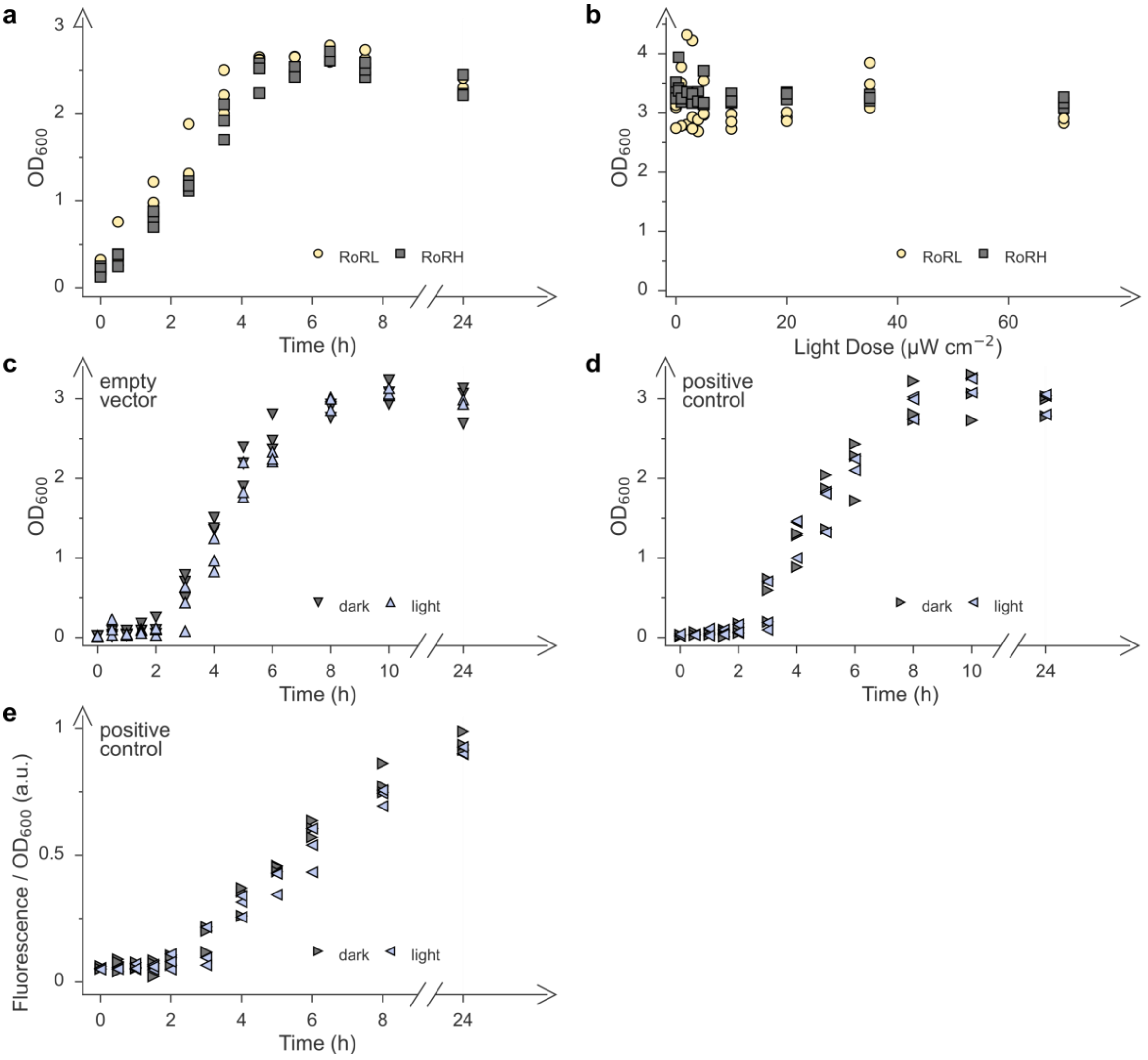

Influence of blue-light exposure on bacterial growth and *Ds*Red reporter-gene expression. **a**, Following exposure to 470-nm light (onset at time zero), the optical density at 600 nm (OD_{600}) of the bacterial cultures from Fig. 3a increased over time in sigmoidal manner. Symbols denote three independent biological replicates each of bacteria harboring the RoRL (yellow circles) and RoRH (grey squares) circuits, respectively. **b**, The OD_{600} values of the bacteria from Fig. 3b that harbor the RoRL or RoRH systems were determined after 24 h incubation at varying intensities of 470-nm light. The readings were similar across both systems, all three biological replicates, and all light intensities, indicating that illumination had no adverse effects. **c**, Bacteria harboring an empty pCDF vector were incubated in darkness (grey) or under 60 µW cm^{-2} blue light (470 nm) (light blue). At the indicated times, aliquots were drawn, and their optical densities at 600 nm were determined. The OD_{600} values increased over incubation time sigmoidally with similar kinetics for all three biological replicates and independent of illumination. **d**, As in panel c but for bacteria carrying a positive-control plasmid constitutively

driving *Ds*Red expression. **e**, The *Ds*Red fluorescence of the bacterial cultures from panel d normalized by OD_{600}.

Supplementary Table 1 – Sequences of Riboptoregulator Circuits

Name	Alternative Name	Sequence [a]
RoR0		aataGatcAccTGCTTGCCGGTTCAGCAGCGAGCCGTAAGCCTCACTGCTGGAACTGATAGGAGGAGAtatacc
RoR1	MM1	aataTatcAccTGCTTGCCGGTTCAGCAGCGAGCCGTAAGCCTCACTGCTGGAACTGATAGGAGGAGAtatacc
RoR2	IL1	aataGatcTccTGCTTGCCGGTTCAGCAGCGAGCCGTAAGCCTCACTGCTGGAACTGATAGGAGGAGAtatacc
RoR3	BL1	aataGatcAccTCTTGCCGGTTCAGCAGCGAGCCGTAAGCCTCACTGCTGGAACTGATAGGAGGAGAtatacc
RoR4	BL2	aataGatcAccTGCTTGCCGGTTCAGCAGCGAGCCGTAAGCCTCACTGCTGAACTGATAGGAGGAGAtatacc
RoR5	IL2	aataGatcAccTGCTTGCCGGTTCAGCAGCGAGCCGTAAGCCTCGCTGCTGGAACTGATAGGAGGAGAtatacc
RoR6	IL3	aataGatcAccTGCTTGTCGGTTCAGCAGCGAGCCGTAAGCCTCACTGCTGGAACTGATAGGAGGAGAtatacc
RoR7	58.14	aataGatcAccTGCTTGCCGGTTCAGCAGCGAGCGATAAGCCTCACTGCTGGAACTGATAGGAGGAGAtatacc
RoR8	58.16	aataGatcAccTGCTTGCCGGTTCAGCAGCGAGCCATAAGCCTCACTGCTGGAACTGATAGGAGGAGAtatacc
RoR9	58.20	aataGatcAccTGCTTGCCGGTTCAGCAGCGAGCCGGAAGCCTCACTGCTGGAACTGATAGGAGGAGAtatacc
RoR10	Δ1	aataGatcAccTGCTTGCGGTTCAGCAGCGAGCCGTAAGCCTCACTGCTGGAACTGTAGGAGGAGAtatacc
RoR11	Δ2	aataGatcAccTGCTTCGGTTCAGCAGCGAGCCGTAAGCCTCACTGCTGGAACTGAGGAGGAGAtatacc
RoR12	Δ3	aataGatcAccTGCTTGGTTCAGCAGCGAGCCATAAGCCTCACTGCTGGAACTAGGAGGAGAtatacc
RoR13	Δ4	aataGatcAccTGCTTGTTCAGCAGCGAGCAATAAGCCTCACTGCTGGAACAGGAGGAGAtatacc
RoRH	RoR0_ep60	aataGatcAccTGCTAGCCGGTTCAGCAGCGAGCCGTAAGCCTCACTGCCGGAACTGATAGGAGGAGAtatacc
RoR14	RoR12_ep25	aataGatcAccTGCTTGGTTCAGCAGCGAGCCATAAGCCACACTGCTGGAACTAGGAGGAGAtatacc
RoR15	RoR0_M1	aataGatcAccTGCTaGCCGGTTCAGCAGCGAGCCGTAAGCCTCACTGCTGGAACTGATAGGAGGAGAtatacc
RoR16	RoR0_M2	aataGatcAccTGCTTGCCGGTTCAGCAGCGAGCCGTAAGCCTCACTGCcGGAACTGATAGGAGGAGAtatacc
RoR17	RoR12_M3	aataGatcAccTGCTTGGTTCAGCAGCGAGCCATAAGCCTCACTGCcGGAACTAGGAGGAGAtatacc

RoRL	RoR12_M4	aataGatcAccTGCTAGGTTCAGCAGCGAGCCTTAAGCCTCACTGCTGGAACTAGGAGGAGAtatacc
RoR18	RoR12_M5	aataGatcAccTGCTTGGTGCAGCAGCGCGCCATAAGCCGCACTGCTGGtACTAGGAGGAGAtatacc
RoR19	RoR12_M6	aataGatcAccTGCTTGGTTCAGCAGCGAGCCAAAAGCCTCACTGCTGGAACTAGGAGGAGAtatacc
RoR20	RoR12_M7	aataGatcAccTGCTAGGTTCAGCAGCGAGCCTTAAGCCACACTGCTGGAACTAGGAGGAGAtatacc

[a]: The PAL aptamer is highlighted in light blue (stem region) and dark blue (loop region). The Shine-Dalgarno-sequence is marked in grey.

Supplementary Table 2 – Predicted Free Energy of RNA Structures within Riboptoregulator Circuits

	Minimum Free Energy (kJ mol^{-1}) [a]	
**Name**	***cis*-RNA [b]**	**PAL aptamer [c]**
RoR0	-112.3	-30.6
RoR1	-125.7	-30.6
RoR2	-134.9	-30.6
RoR3	-128.2	-30.6
RoR4	-128.2	-30.6
RoR5	-134.9	-30.6
RoR6	-133.2	-30.6
RoR7	-116.5	-6.7
RoR8	-112.3	-20.5
RoR9	-112.3	-44.4
RoR10	-127.8	-72.1
RoR11	-123.2	-30.6
RoR12	-111.5	-27.2
RoR13	-108.1	-17.6
RoRH	-113.5	-30.6
RoR14	-94.3	-27.2
RoR15	-119.8	-30.6
RoR16	-106.0	-30.6
RoR17	-105.2	-27.2
RoRL	-121.5	-27.2
RoR18	-117.7	-34.8
RoR19	-111.5	-31.4
RoR20	-104.3	-27.2

[a]: Calculated with RNAfold (2).

[b]: Determined for the entire *cis*-RNA as shown in Suppl. Table S1.

[c]: Determined for the isolated PAL aptamer, highlighted in light and dark blue in Suppl. Table S1.

Supplementary References

1. Ranzani,A.T., Wehrmann,M., Kaiser,J., Juraschitz,M., Weber,A.M., Pietruschka,G., Gerken,U., Mayer,G. and Möglich,A. (2022) Light-Dependent Control of Bacterial Expression at the mRNA Level. *ACS Synth. Biol.*, **11**, 3482–3492.

2. Gruber,A.R., Lorenz,R., Bernhart,S.H., Neuböck,R. and Hofacker,I.L. (2008) The Vienna RNA websuite. *Nucleic Acids Res.*, **36**, W70-74.
